# A meta-analysis of the efficacy and safety of accelerated partial breast irradiation versus whole-breast irradiation for early-stage breast cancer

**DOI:** 10.1186/s13014-021-01752-2

**Published:** 2021-02-02

**Authors:** Xiaoyong Xiang, Zhen Ding, Lingling Feng, Ning Li

**Affiliations:** 1 Department of Radiation Oncology, National Cancer Center/National Clinical Research Center for Cancer/Cancer Hospital & Shenzhen Hospital, Chinese Academy of Medical Sciences and Peking Union Medical College, Shenzhen, 518116 China; 2 Department of Radiation Oncology, National Cancer Center/National Clinical Research Center for Cancer/Cancer Hospital, Chinese Academy of Medical Sciences and Peking Union Medical College, Beijing, 100021 China

**Keywords:** Accelerated partial breast irradiation, Whole-breast irradiation, Breast cancer, Breast-conserving surgery, Meta-analysis

## Abstract

**Objective:**

This meta-analysis evaluated the efficacy and safety of accelerated partial breast irradiation versus whole-breast irradiation for early-stage breast cancer after breast-conserving surgery.

**Materials and methods:**

A systematic search of PubMed, Embase, and the Cochrane libraries was performed according to the PRISMA statement the last 10 years to April 7, 2020 to identify the randomized controlled trials of APBI versus WBI for treating patients with early-stage breast cancer. Two independent observers evaluated the identified studies. The obtained data were analyzed using the RevMan 5.3 software.

**Results:**

A total of 10 randomized controlled trials involving 15,500 patients with early-stage breast cancer were selected according to the inclusion and exclusion criteria and included in this meta-analysis. In this meta-analysis, we included ten studies that reported local recurrence and found significant differences in local recurrence rates (HR = 1.46; 95% CI 1.20–1.79, *P* = 0.0002). Further analysis showed that this difference may be related to the choice of treatment methods. No differences in distant metastasis, breast cancer deaths, contralateral breast cancer, disease-free survival, and overall survival rates were observed between WBI and APBI groups. There was no significant difference in late toxicity, cosmetic outcomes and quality of life between the two groups, the compliance and tolerance of the patients were well. Compared to whole breast irradiation, accelerated partial breast irradiation significantly reduced serious (≥ grade 2) early toxicities, especially regarding acute skin toxicity.

**Conclusions:**

The analysis showed that patients receiving APBI had a higher local recurrence rate, but no differences in distant metastasis, breast cancer deaths, contralateral breast cancer, disease-free survival, and overall survival rates.

## Introduction

Breast cancer was the most frequently diagnosed cancer and the most frequent cause of death from cancer in women [1]. Breast-conserving surgery combined with whole-breast irradiation (WBI) has been the gold standard therapy for patients with early-stage breast cancer, which can yield cancer outcomes comparable to mastectomy [2, 3]. WBI is usually delivered once per day over several consecutive weeks, making access to effective radiotherapy problematic for women with some socioeconomic barriers [4–6]. As most patients with early-stage breast cancer are cured of their disease, long-term toxicities become more and more critical [2].

Recurrence patterns after breast-conservation surgery suggest that most local recurrences occur predominantly at or near the breast tissue adjacent to the post-excision lumpectomy cavity [7, 8]. Accelerated partial breast irradiation (APBI) only irradiates the tumor bed in one week or less, which is a very favorable treatment that can reduce the burden of care and make it more likely to be accepted by patients [9]. Moreover, due to the smaller irradiation range of APBI, it is expected to reduce toxicity and improve cosmetic effect and quality of life compared with whole-breast irradiation [10].

APBI technology was introduced into clinical practice in the 1990s [11, 12], several different techniques have been developed, including intraoperative irradiation (IORT) with electrons or photons, multicatheter or single brachytherapy, and external beam radiotherapy using intensity-modulated radiotherapy (IMRT) or three-dimensional conformal radiotherapy (3DCRT). Current treatment guidelines [13, 14] and previous meta-analysis of randomized trials [15, 16] regarding APBI mainly address brachytherapy and IORT, these techniques are resource-intensive and invasive, requiring specialized radiotherapy delivery systems and surgical procedures. However, external beam radiotherapy such as 3DCRT and IMRT are noninvasive and only need the widely used CT planning system and linear accelerator. Recently, a large randomized phase 3 trial main using 3DCRT [17] and another [18] using 3DCRT or IMRT in the APBI arm have been officially published, but their results are still controversial. APBI is only applicable to highly selected breast cancer with low-risk factors and has not been widely used in clinical practice.

Here, we performed a systematic review and meta-analysis of all those published randomized studies adopting the APBI for early-stage breast cancer with the primary aims being LR (local recurrence), NR (regional recurrence), safety, cosmetic efficacy, and long-term survival outcome compared with WBI.

## Methods

### Literature search strategy

Before starting the meta-analysis, all the researchers looked at the Prospero, and used the Prisma-P tool to prepare the meta-analysis. A bibliographical search was performed of PubMed, Embase, and the Cochrane Library according to the PRISMA statement the last 10 years to April 7, 2020. The main keywords used for the search were ‘breast cancer’, ‘breast neoplasms’, ‘accelerated partial breast irradiation’, ‘APBI’, ‘whole breast irradiation’, ‘WBI’. Searches were limited to human and English language studies. Retrieve the relevant studies manually if necessary.

### Selection criteria

The eligibility criteria of the study are as follows: (1) Patients diagnosed with early-stage breast cancer; (2) Two comparison groups, one group receiving accelerated partial breast radiotherapy and the other group receiving whole breast radiotherapy; (3) At least local recurrence rate data are reported or reported other outcomes (such as OS, DFS, distant metastasis rate, NR, toxicity, cosmetic effect); (4) Randomized controlled trials (RCTs); (5) Language restrictions in English; (6) the sample size of the study was more than 50 cases. Exclusion criteria included the following: (1) Reviews and meta-analyses, abstracts, case reports, and lectures; (2) The clinical diagnosis of patients is unclear; (3) Incorrect or incomplete data that unable to extract data from other relevant studies; (4) Duplicate publications. In the case of overlapping studies, only the most informative or latest researches were included in the analysis. Articles that fulfilled the inclusion and exclusion criteria were retrieved for full-text evaluation and extracted data from the context of the article.

### Data extraction and quality assessment

After reviewing the full texts of eligible studies, two independent investigators (Xiaoyong Xiang and Zhen Ding) extracted the data and cross-checked all the results. Potential differences in selecting articles and extracting data were resolved with a third reviewer (Ning Li). The extracted variables include general study characteristics (e.g., author, year of publication, study period, median follow-up, number of patients), clinical characteristics (e.g., median age, tumor stage, ER+ or Her-2+ rate, high-grade tumors, histology subtype, pre-menopausal patients rate), treatment characteristics (e.g., radiotherapy technique, RT dosage), short- and long-term outcomes (e.g., local recurrence, regional recurrence, distant metastasis; breast cancer mortality; HR for DFS, OS and LR [if available]; the rate of OS, DFS, LR, NR at 5, 8, 10, and 12 years; cosmetic outcome rating (fair + poor), and toxicities (e.g., Late or acute skin toxicity, fatty necrosis, induration or fibrosis). Because all the included studies are randomized, the methodological quality of the studies was evaluated with the Jadad score. Each study with Jadad scores ≤ 3 was considered a low-quality study, whereas studies with Jadad scores > 3 were considered high-quality. The results of the quality assessment are summarized in Table 1.

**Table 1:** The main detailed characteristics of the included studies

Author/year	Follow-up (years)	Study Period	Sample Size (n, APBI/WBI)	Jadad score	Age (years)	Stage	Receptor status	Premen-opausal	Histology	Treatment arms
Jayant 2020 [23]	9	2004–2012	581/572	5	63	0 ~ II	ER + :98% HER2 + :5.5%	NR	Grade 3: 5.6% IDC:100%	APBI: 50 kV energy X-rays IORT 20 Gy WBI:EBRT40–56 Gy/15–25F ± boost10–16 Gy/5–8F
Vicini 2019 [24]	10.2	2005–2013	2036/2089	5	54	0 ~ II	ER + :81% HER2 + :NR	39%	Grade 3: 26.8% DCIS: 24.5%	APBI: HDR brachytherapy 34 Gy or 3DCRT 38.5 Gy/10F, BID WBI: EBRT 50 Gy/25F ± Boost 10 Gy/5 F at least
Whelan 2019 [18]	8.6	2006–2011	1070/1065	5	61	0 ~ II	ER + :90% HER2 + :4.7%	NR	Grade 3: 12.9% DCIS: 18%	APBI: 3DCRT or IMRT 38.5 Gy/10F,BID WBI: External beam 42.5 Gy/16F or 50 Gy/25F ± Boost 10 Gy/5F
Coles 2017 [25, 26]	6.1	2007–2010	669/674	5	62	I ~ II	ER + : 95.1% HER2 + : 4%	NR	Grade 3: 9.5% IDC: 85.4%	APBI: IMRT 40 Gy/15F WBI: IMRT 40 Gy/15F
Strnad 2016 [19]	6.6	2004–2009	633/551	7	62	0 ~ IIA	ER + :91.4% HER2 + :NR	16.9%	Grade 3: 8.4% IDC: 74.1%	APBI: HDR brachytherapy 32 Gy/8F or 30.1 Gy/7F BID PDR brachytherapy 50 Gy/0.6–0.8 Gy/per h pulses,24 h/day WBI: (4–10MV) photon beams 50.0–50.4 Gy /25–28F ± Boost 10 Gy/5F
Livi 2015 [27, 28]	5	2005–2013	260/260	5	60–69	I ~ II	ER + : 95.4% HER2 + :3.6%	NR	Grade 3:11.4% IDC: 57.5% DCIS: 10.6%	APBI: IMRT 30 Gy/5F WBI: IMRT 50 Gy/25F + Boost 10 Gy/5F
Vaidya 2014 [29, 30]	2.4	2000–2002	1679/1696	5	61–70	I ~ IIIA	ER + :93% HER2 + :11.6%	NR	Grade 3: 15.2% IDC: 100%	APBI: 50 kV energy X-rays IORT 20 Gy WBI: EBRT40–56 Gy/15–25F ± boost10–16 Gy/5–8F
Polgár 2013 [31]	10.2	1998–2004	128/130	4	58.5	I ~ II	ER + :88.7% HER2 + :NR	21.3%	Grade 3: 0% IDC: 81.8	APBI: HDR brachytherapy 36.4 Gy/7F, BID; Protocol allowed 50 Gy limited field electron beam if patients unsuitable for brachytherapy WBI: Telecobalt or 6–9MV photon beams using wedged tangential fields 42–50 Gy/2 Gy per day
Veronesi2013 [32]	5.8	2000–2007	651/654	5	60–69	I ~ II	ER + : 90.8% HER2 + :3.4%	NR	Grade 3: 21.7% IDC: 80.2%	APBI: Electron IORT 21 Gy WBI: EBRT 50 Gy/25F + Boost 10 Gy/5F
Rodriguez 2013[33]	5	NR	51/51	4	68.6	I ~ II	ER + : 98% HER2 + : 1%	0%	Grade 3: 0% ILC excluded	APBI: 3DCRT 37.5 Gy/10F,BID WBI: 3DCRT 48 Gy/24F ± Boost 10 Gy/5F

*IMRT* intensity-modulated radiation therapy, *IORT* intraoperative irradiation, *3DCRT* three-dimensional conformal radiation therapy, *EBRT* whole-breast external beam radiotherapy, *HDR* high dose rate, *WBI* whole-breast irradiation, *APBI* accelerated breast irradiation, *ER* estrogen receptor, *HER2* human epidermal growth factor receptor 2, *IDC* invasive duct carcinoma, *DCIS* ductal carcinoma in situ, *ILC* invasive lobular carcinoma, *BID* Boost tumor bed, two fractions per day, *NR* not reported

### Statistical analysis

The primary endpoint was the LR percentage in the APBI arm. Secondary endpoints were NR, breast cancer mortality, cosmetic outcome, distant metastasis, OS, DFS, and toxicity. Odds ratio (OR) and 95% confidence interval (95% CI) for count data, HR and 95% CI for OS and DFS were pooled into formal meta-analyses. Using the Cochrane Q test and the I^2^ statistics to evaluate the heterogeneity between studies. If heterogeneity was present (*P* < 0.1, I^2^ > 50%), the statistical pooling of effect measures was based on the random-effect model. Otherwise, a fixed-effect model was employ. Subgroup analysis was performed according to radiotherapy techniques (TPS vs. Not TPS). The analysis results were shown in the forest maps, and the potential heterogeneity was identified by sensitivity analysis.

Subsequently, publication bias was assessed using Begg’s and Egger’s regression asymmetry tests. Statistical analyses were performed using RevMan 5.3 (The Cochrane Centre), and a difference with P value < 0.05 was considered statistically significant.

## Results

**Fig. 1:**
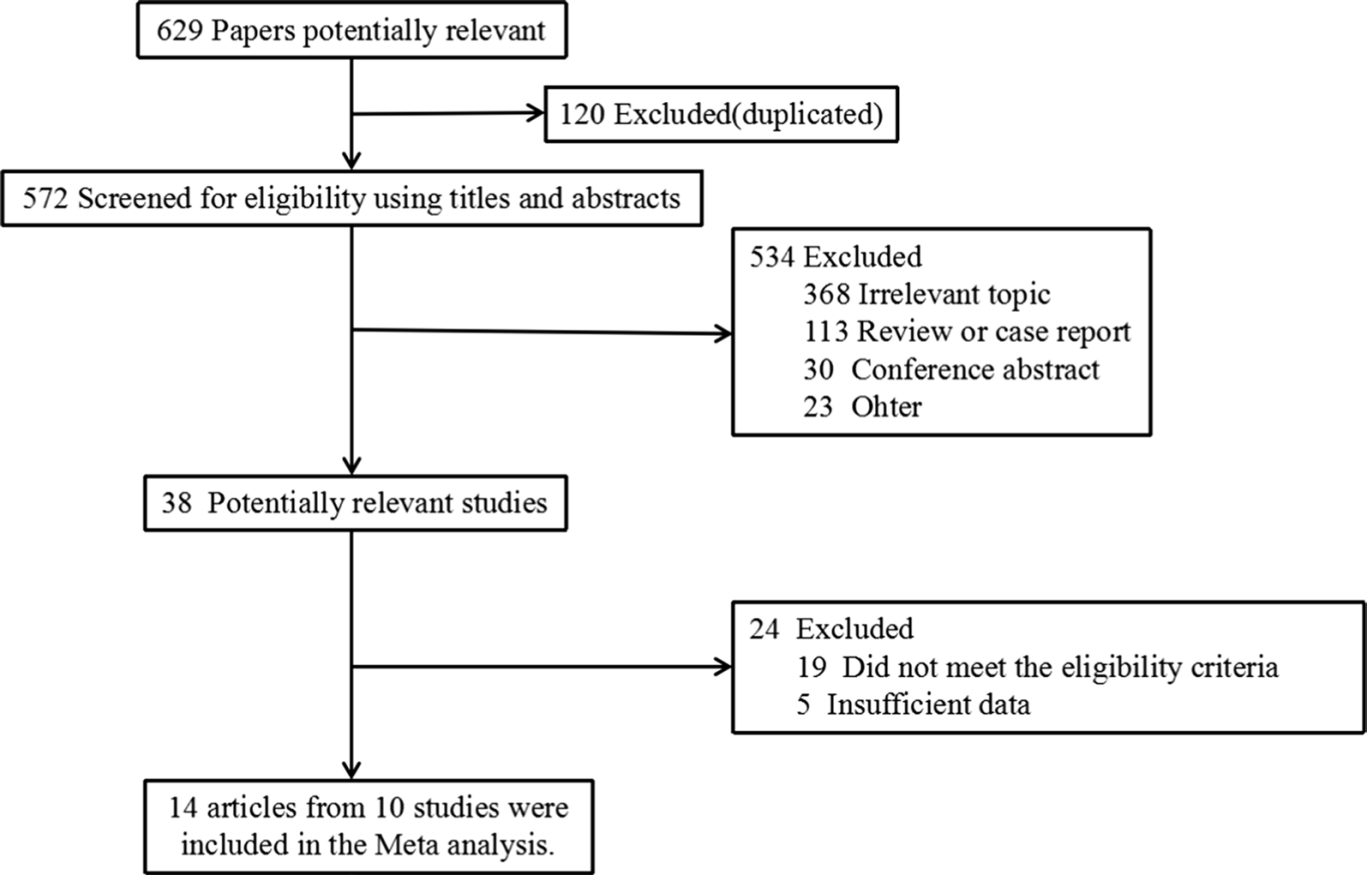
Flow diagram of included studies

After screening 692 records, a total of 38 studies were considered to meet the criteria for inclusion in the systematic review potentially. Ultimately, 14 publications reporting on outcomes from 10 studies were included in the analysis. The GEC-ESTRO study reported on efficacy [19], early toxicity and patient compliance [20], late side-effects and cosmetic results [21], and quality-of-life results in different publications [22]. Studies were published in 2013–2020, 15,500 patients with early-stage breast cancer, including 7758 in the APBI group and 7742 in the WBI group. The flowchart of the literature search and selection process is shown in Fig. 1, while the characteristics of the eligible studies and main outcomes are summarized in Tables 1 and 2.

**Table 2:** Main long-term outcomes

Author/year	Group	5–8–10–12 year DFS	5–8–10–12 year OS	5–8–10–12 year LR	5–8–10 year NR	Breast cancer deaths	Distant metastasis	Contralateral breast cancer	Second primary cancers
Jayant 2020 [23]	APBI WBI	NR	96.70%/–/88.62%/83.13% 97.69%/–/87.77%/84.72%	3.96%/–/7.2%/7.2% 1.05%/–/2.8%/3.5%	NR	3.61% 3.0%	1.0% 1.4%	NR	NR
Vicini 2019 [24]	APBI WBI	–/–/78.1%/– –/–/79.7%/–	–/–/90.6%/– –/–/91.3%/–	–/–/4.6%/– –/–/3.9%/–	NR	2.3% 2.2%	2.4% 3.1%	3.0% 3.5%	9% 10%
Whelan 2019 [18]	APBI WBI	96.4%/94.9%/–/ 96.8%/95.4%/–/	96.2%/93.6%/–/– 97%/94.3%/–/–	2.3%/3%/–/– 1.7%/2.8%/–/–	–/0.4%/– –/0.2%/–	1.7% 1.5%	1.9% 1.7%	2.7% 3.6%	10.6% 9.0%
Coles 2017 [25, 26]	APBI WBI	NR	NR	0.5%/–/–/– 1.1%/–/–/–	0.3%/–/– 0.1%/–/–	1.5% 1.3%	1.8% 1.9%	1.9% 1.8%	5.5% 7.0%
Strnad 2016 [19–22]	APBI WBI	95.03%/–/–/– 94.45%/–/–/–	97.27%/–/–/– 95.55%/–/–/–	1·44%/–/–/– 0·92%/–/–/–	0.47%/–/– 0.18%/–/–	0.6% 0.7%	0.8% 0.9%	0.8% 0.9%	5.5% 4.0%
Livi 2015 [27, 28]	APBI WBI	NR	99.4%/–/–/– 96.6%/–/–/–	1.5%/–/–/– 1.4%/–/–/–	1.5%/–/– 1.9%/–/–	0.4% 1.2%	1.2% 1.5%	1.2% 2.7%	NR
Vaidya 2014 [29, 30]	APBI WBI	NR	NR	3.3%/–/–/– 1.3%/–/–/–	0.5%/–/– 0.3%/–/–	1.2% 0.9%	0.5% 0.4%	NR	NR
Polgár 2013 [31]	APBI WBI	88.8%/–/85.3%/– 90.5%/–/83.6%/–	–/–/79.7%/– –/–/82.1%/–	4.0%/–/5.9%/– 3.3%/–/5.1%/–	1.6%/–/2.5% 1.7%/–/1.7%	NR	5.5% 8.5%	7.0% 6.2%	12.5% 10.8%
Veronesi2013 [32]	APBI WBI	NR	96.8%/–/–/– 96.9%/–/–/–	4.4%/–/–/– 0.4%/–/–/–	1.0%/–/– 0.3%/–/–	3.5% 3.1%	5.1% 5.4%	1.2% 2.0%	4.3% 5.4%
Rodriguez 2013 [33]	APBI WBI	NR	NR	0%/–/–/– 0%/–/–/–	0%/–/– 0%/–/–	0% 0%	0% 0%	NR	5.5% 7.0%

*OS* overall survival, *DFS* disease-free survival, *LR* local recurrence, *RR* regional recurrence, *NR* no reported, *Adverse cosmesis* physician-scored cosmetic results fair and poor, *Second primary cancers* had a second primary tumor in the contralateral-breast or a second tumor at a site other than the breast

### Local recurrence

**Fig. 2:**
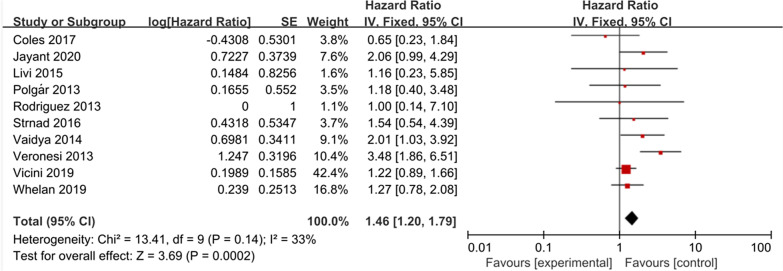
Forest plot of the Hazard Ratio of the local recurrence (LR)

Ten eligible studies had local recurrence data, and the studies included 7,758 patients in the APBI group and 7,742 patients in the WBI group. There was a significant difference between the two groups (HR = 1.46, 95% CI 1.20–1.79, *P* = 0.0002; heterogeneity *P* = 0.14, I^2^ = 33%, Fig. 2). The analysis showed that patients receiving APBI had a higher local recurrence rate.

### Regional recurrence

**Fig. 3:**
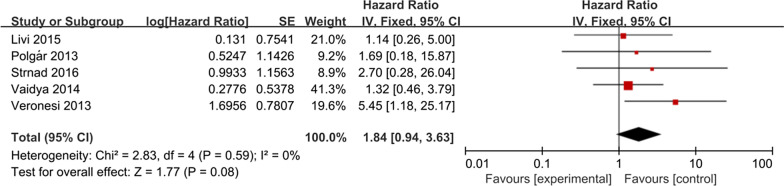
Forest plot of the hazard ratio of the regional recurrence

Five studies can extract HR data of regional recurrence. Meta-analysis showed that there was no statistical significance between the two groups, but the WBI group had a trend to reduce the regional recurrence risk (HR = 1.84; 95% CI 0.94–3.63, *P* = 0.08, Fig. 3). The included studies had no significant heterogeneity (*P* = 0.59, I^2^ = 0%).

### Distant metastasis

**Fig. 4:**
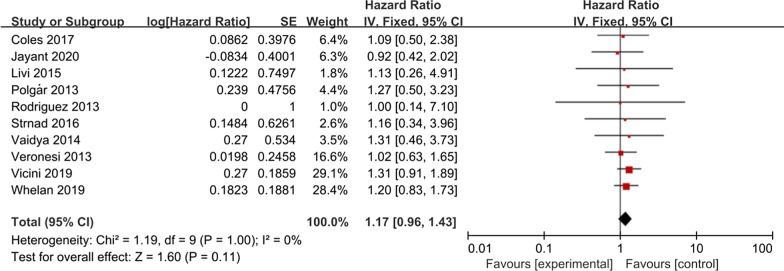
Forest plot of the Hazard Ratio of the distant metastasis

Ten studies reported the impact of APBI/WBI on distant metastasis.The meta-analysis showed that there was no significant difference between APBI group and WBI group (HR = 1.17; 95% CI 0.96–1.43, *P* = 0.11; heterogeneity *P* = 1.00, I^2^ = 0%, Fig. 4).

### Overall survival

**Fig. 5:**
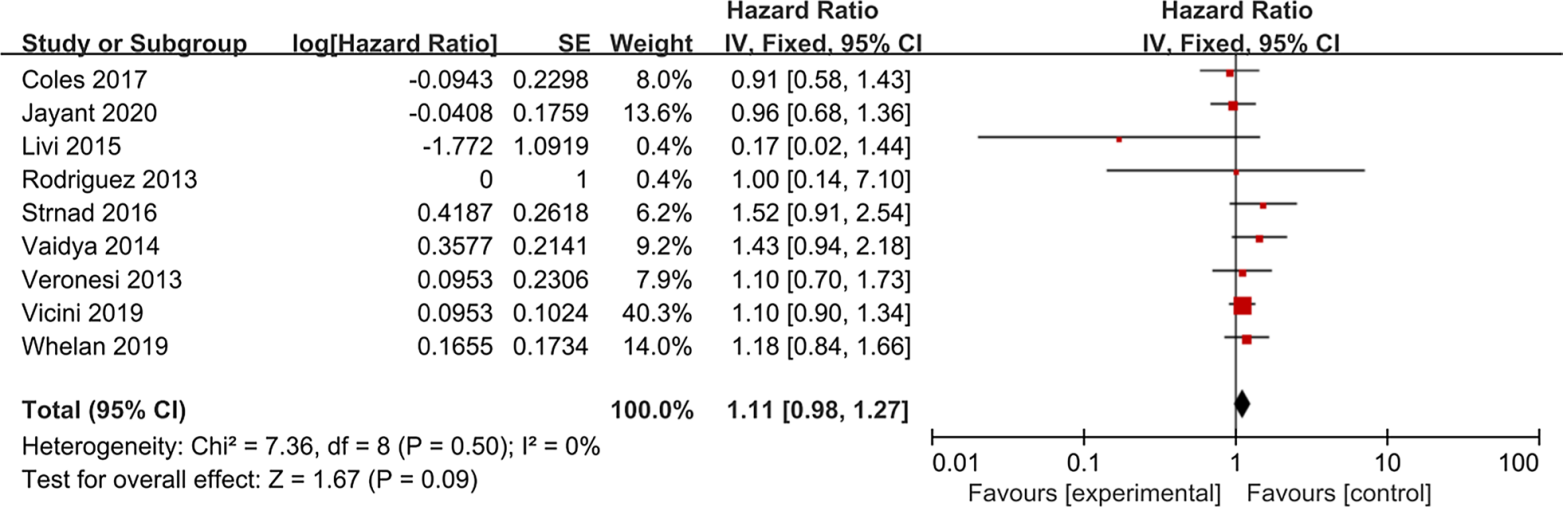
Forest plot of the hazard ratio of the overall survival

Nine studies, including 15,242 patients, compared the OS rate of APBI versus WBI in patients with early-stage breast cancer. The heterogeneity test results were *P* = 0.50 and I^2^ = 0%, indicating a low risk of heterogeneity; the fixed-effects model was then used. The forest plots of the meta-analysis showed that there was no significant difference in overall survival rate between the APBI group and WBI group (HR = 1.11, 95% CI 0.98–1.27, *P* = 0.09; heterogeneity *P* = 0.50, I^2^ = 0%, Fig. 5).

### Disease-free survival

**Fig. 6:**
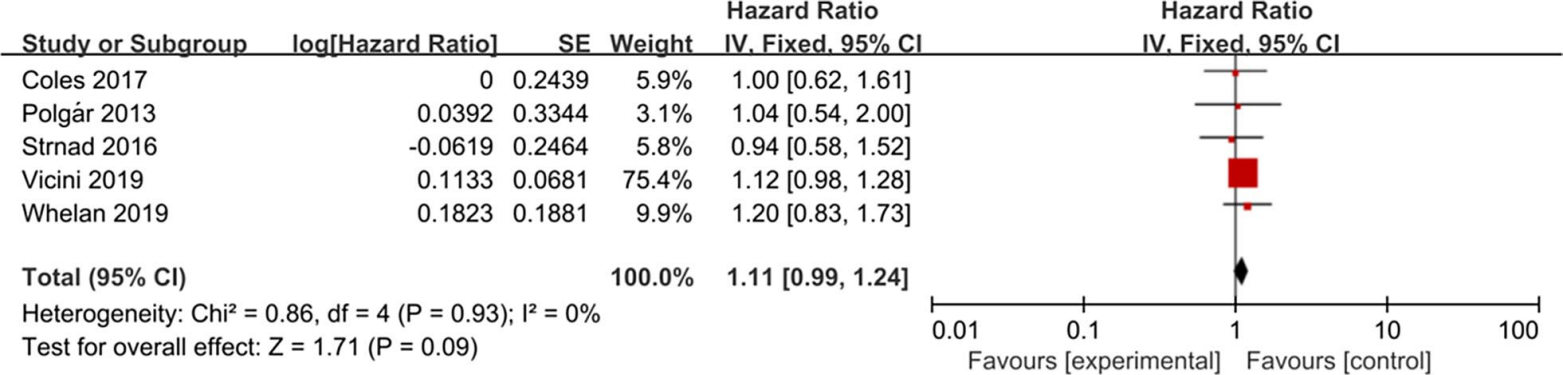
Forest plot of the hazard ratio of the disease-free survival

There were five eligible studies had regional recurrence data; these studies included 4536 patients in the APBI group and 4509 patients in the WBI group. Subsequent analysis of these studies revealed that there was no significant difference in disease-free survival rate between APBI and WBI groups (HR = 1.11, 95% CI 0.99–1.24, *P* = 0.09, Fig. 6). The included studies had no significant heterogeneity (*P* = 0.93, I^2^ = 0%).

### Breast cancer deaths

**Fig. 7:**
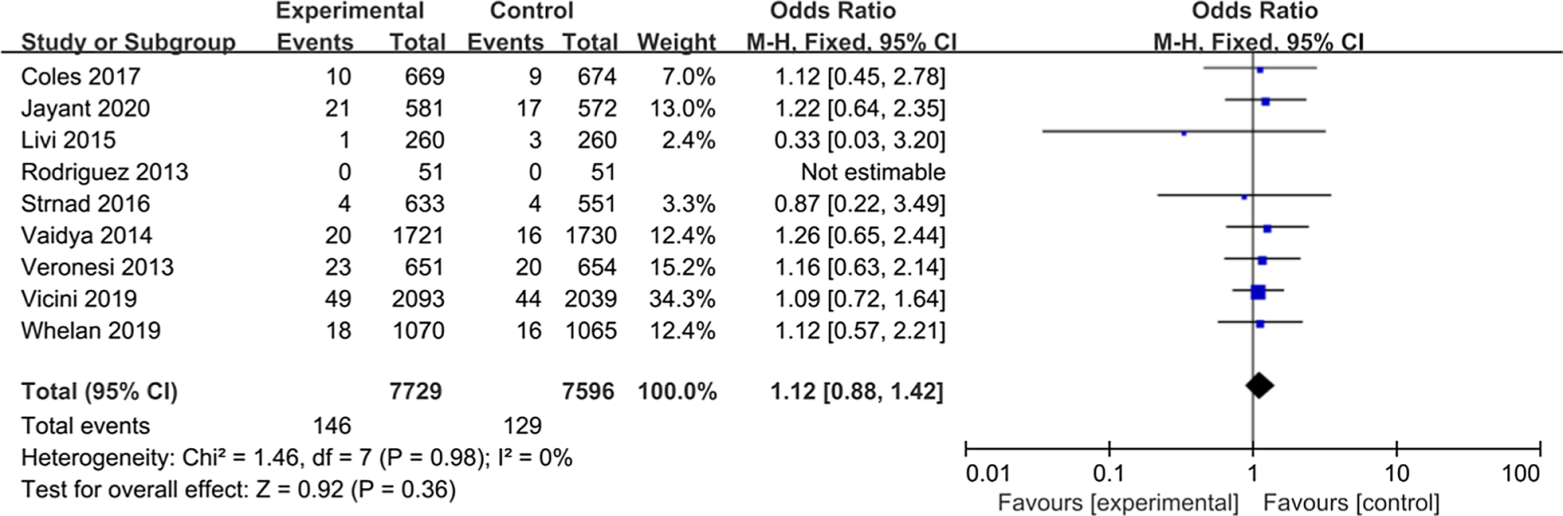
Forest plot of the Odds Ratio of breast cancer deaths

Breast cancer death was reported for nine studies; there were 7729 patients in the APBI group and 7596 patients in the WBI group. Subsequent analysis of these studies revealed no significant difference in breast cancer mortality between two groups (OR = 1.12, 95%CI 0.88–1.42, *P* = 0.36, Fig. 7). The included studies had no significant heterogeneity (*P* = 0.98, I^2^ = 0%).

### Contralateral breast cancer

**Fig. 8:**
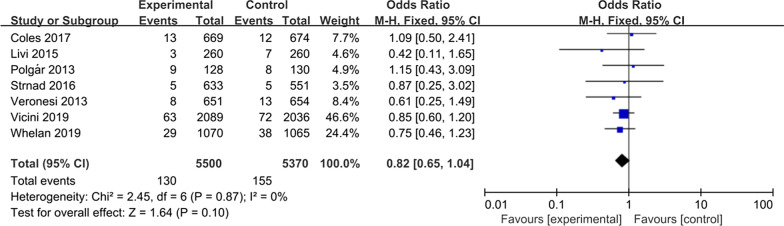
Forest plot of the odds ratio of the contralateral breast cancer

Seven studies were available for comparative analysis of contralateral breast cancer, including 5,500 patients in the APBI group, and 5,370 patients in the WBI group. Meta-analysis showed that there was no significant difference in the rate of contralateral breast cancer between the two groups (OR = 0.82, 95% CI 0.46–1.23, *P* = 0.10, Fig. 8) and no heterogeneity between the included studies (*P* = 0.87, I^2^ = 0%).

### Toxicity, cosmetic outcomes and quality of life

Eight, five, and two studies respectively reported the major toxicity, cosmetic effects, and quality of life. Because their endpoints and criteria were not uniform, we presented a descriptive analysis of their results (Table 3). In addition, a sub-study of the TARGIT-A trial found that patients treated with APBI have similar self-reported cosmetic outcome but better breast-related quality of life outcomes than patients treated with WBI [46], and it is found that for cosmetic appearance and other results, the patient's point of view is the most important [47].

**Table 3:** Main toxicity and cosmetic outcomes (fair/poor)

Author/year	Group	Cosmetic outcome (fair/poor)		Induration or Fibrosis Grad ≥ 2	Telangie-ctasia Grad ≥ 2	Breast pain Grad ≥ 2	Fatty necrosis Grade ≥ 2	Acute Skin toxicity Grade ≥ 2	Late skin toxicity Grade ≥ 2	Acute toxicity	Late toxicity	CTCAE /RTOG Grade ≥ 2	Acute pneumonitis Grade ≥ 2
		Physician-rated	Self-rated										
Vicini 2019 [17]	APBI WBI											54.2% 66.1%	
Whelan2019 [18]	APBI WBI	32% 16%(5-years)	30% 18%(5-years)	22.9% 4.6%	9.3% 3.7%	4.8% 1.9%	2.7% < 0.5%			9.5% 30.8%	32.3% 13.3%		
Strnad 2016 [19–22]	APBI WBI	7% 10%(5-years)	8% 9%(5-years)	6.4% 4.6%	4.1% 4.6%	8.4% 11.9%	1.4% 1%	2.2% 43.5%	3.92% 6.1%				
Livi 2015 [27, 28]	APBI WBI	0% 0.8%						1.9% 37.7%	0% 0.8%				
Vaidya 2014 [29, 30]	APBI WBI								0.23% 0.75% (Grade3/4)			3.3% 3.9% (Grade3/4)	
Polgár 2013 [31]	APBI WBI	19.2% 37.1%											
Veronesi 2013 [32]	APBI WBI						4.74% 2.43%	1.08% 7.77% (Any acute)	1.29% 1.21% (Any chronic)				
Rodriguez 2013 [33]	APBI WBI		0% 0%					17.6% 74.5%	0% 0%				0% A single case

### Publication bias

There was no significant publication bias in the meta-analysis of all effects. For the local recurrence meta-analysis, there was no evidence of publication bias, and neither Begg nor Egger test was significant (*P* = 0.59 and 0.25).

## Discussion

Adjuvant whole-breast irradiation after breast-conserving surgery can significantly reduce the risk of local and regional recurrences and has shown a positive influence on overall survival especially for patients with an intermediate to high absolute risk for local recurrences compared to lumpectomy alone, which has become the standard treatment for early-stage breast cancer [3, 34, 35]. Although adjuvant radiotherapy after breast-conserving surgery is crucial, many studies have shown that local recurrences frequently occur near the primary tumor location. Consequently, it is considered that radiotherapy’s main benefits arise from irradiating partial breast around the surgical cavity [7, 36, 37].

Accelerated partial breast irradiation is performed by aiming radiation delivery to the surgical cavity and its surrounding 1–2 cm breast tissue, which is considered to be the tissue with the highest risk of tumor cell residue after breast-conserving surgery [17, 18]. There are numerous techniques for APBI, including external beam-based APBI; IORT with either gamma-rays, photons or electrons; brachytherapy (interstitial or intracavitary) [38]. Commonly fractionation schemes include 38 Gy in 10 fractions with external beam-based APBI, 20–21 Gy in one fraction with IORT, or 34 Gy in 10 fractions for brachytherapy [38–40]. Because the irradiation range is narrowed and the α/β ratio of breast cancer cells is lower than that of other tumors [41], accelerated large-division irradiation does not significantly increase acute or late radiotherapy responses. At the same time, APBI shortens the total treatment time, saves medical resources, and reducing patients' treatment costs and waiting time, which is of great economic significance [9, 42].

Although APBI has many advantages, there is still no unified standard for its techniques, indications, and fractionation schemes. At present, several societies have published guidelines to define whether patients can perform APBI: those of ASTRO (American Society for Radiation Oncology), GECESTRO (European Society for Radiotherapy and Oncology), and ABS (American Brachytherapy Society)[13, 43, 44]. In addition to consistent standards regarding age ≥ 50 years, negative node status, and absence of lymphovascular space invasion. There is no general consensus on other criteria such as tumor size, molecular typing, lymph node invasion, and other characteristics [13, 43, 44]. Consequently, the current guidelines recommend that patients receiving APBI should be carefully selected according to their clinical characteristics.

We reviewed a previously published meta-analysis that included seven trials for a total of 7452 patients [16]. This meta-analysis includes a study published in 1993 on the use of single-electron beams for APBI irradiation [11]. We believe that the technical conditions of radiotherapy used at that time are significantly different from those used later, so we have only included the latest ten studies. This meta-analysis showed that there was a significant difference in the 5-year local recurrence rate between the two groups (HR = 4.5, 95% CI 1.78–11.61, *P* = 0.002). There was no significant difference in regional recurrence, systemic recurrence, overall survival, or mortality rates between the two groups. The two groups’ side effects and cosmetic effects were similar, but intraoperative radiotherapy seemed to have greater acute side effects [16].

A recent meta-analysis, the literature search that ended in January 2018, included a total of eleven publications reporting nine studies findings, but two of them were informal data from conference summaries [45]. Our study includes accurate data that have been officially published in both studies, as well as additional research on long-term follow-up survival data for intraoperative radiotherapy. This study used odds ratios (OR) in their meta-analysis of local recurrence, non-breast cancer mortality, overall survival, regional recurrence, contralateral breast cancer, disease-free survival rate, and toxicity. However, HR is the appropriate natural indicator of time-to-event data, and we believe that HR is more accurate than OR in survival analysis. Consequently, HR is extracted as much as possible in our study, and then meta-analysis is performed. Besides, this study performed a subgroup analysis according to radiotherapy techniques, such as EBRT (external beam radiation treatment), brachytherapy, IORT, and other techniques. The subgroup analysis may not be the most appropriate because of each subgroup, including only a small number of studies. However, we performed a subgroup analysis of local recurrence according to whether the patients received therapy with Radiotherapy Treatment Planning System (TPS). There were seven studies in the TPS group and three studies in the Not TPS group.

In this meta-analysis, we included ten studies that reported local recurrence and found significant differences in local recurrence rates (HR = 1.46; 95% CI 1.20–1.79, *P* = 0.0002; heterogeneity *P* = 0.14, I^2^ = 33%). We also note that there is a slight heterogeneity between the included studies, which may be due to the choice of treatment techniques. A total of three studies used IORT, two of which used IOERT (Intraoperative Electron Radiation Therapy)[32], and the other used TARGIT (Targeted intra-operative radiotherapy)[23, 29]. Obviously, 3DCRT, IMRT or brachytherapy based on TPS system have an accurate definition of the target area or dose distribution, while IORT and TARGIT are not involved. Therefore, we performed a subgroup analysis according to whether the patients received therapy with Radiotherapy Treatment Planning System (TPS). The results showed that the studies of the subgroups were homogeneous (*P* > 0.1, I^2^ = 0%). Compared with the total heterogeneity, the heterogeneity of the subgroups was significantly reduced after subgroup analysis, and there was a statistical difference between the subgroups (*P* = 0.002, I^2^ = 90.0%), it is suggested that the main cause of heterogeneity could be the TPS technology. Subset analyses showed that without TPS, APBI could significantly increase LR rate (HR = 2.50, 95% CI 1.69–3.68, *P* < 0.00001; heterogeneity *P* = 0.42, I^2^ = 0%). However, with TPS, there was no significant difference in LR between the APBI group and the WBI group (HR = 1.20, 95% CI 0.95–1.52, *P* = 0.13; heterogeneity *P* = 0.95, I^2^ = 0%). Therefore, we made a assumption that although APBI showed significant disadvantages in local control (similar to the results of previous meta-analyses), the selection of appropriate radiotherapy techniques may eliminate this difference. Although, from the patient's perspective, perhaps the most convenient APBI technique is IORT, which requires only one irradiation during breast-conserving surgery. IORT can not only improve the treatment compliance of patients but also decrease the irradiation of healthy organs and reduce the cost of treatment. However, external beam-based APBI, such as 3DCRT, IMRT radiotherapy are widely available. Multiple randomized trials using this radiation technique have been published and achieved the desired results. Another TPS-based brachytherapy technique predominantly depends on the experience and skills of the treating physician and is only available inexperienced institutions. Consequently, perhaps external beam-based APBI is the most appropriate technology.

No differences in distant metastasis, breast cancer deaths, contralateral breast cancer, disease-free survival, and overall survival rates were observed between WBI and APBI groups. In other words, our study has shown that when using APBI, these outcomes are not worse than WBI. Five studies can extract HR data of regional recurrence, the meta-analysis showed that there was no statistical significance between the two groups, but the WBI group had a trend to reduce the regional recurrence risk (HR = 1.84; 95% CI 0.94–3.63; *P* = 0.08).

Because the endpoints and criteria of toxicity, cosmetic outcomes, and quality of life were not uniform, we only made a descriptive analysis of their results. Compared to WBI, APBI significantly reduced serious (≥ grade 2) early toxicities, especially regarding acute skin toxicity [18, 20, 28, 32, 33]. Although less acute toxicity was observed, the regimen used was associated with an increase in late normal-tissue toxicity and adverse cosmesis in the RAPID trial, which might be related to the twice per day treatment [18]. Two studies reported patients' quality-of-life results; APBI was not associated with worse quality of life than WBI [22, 27]. Overall, there was no significant difference in toxicity, cosmetic outcomes and quality of life between the two groups, the compliance and tolerance of the patients were well.

The main limitations of our meta-analysis are related to the included studies rather than the systematic review itself. Because there are significant differences in times and treatment methods for APBI and WBI, blinding of patients and/or outcome assessors was not possible. However, it should be considered that in this kind of intervention, masking is not possible. In addition, most studies do not have independent data analysts. We believe that the objective results are unlikely to be significantly influenced by the lack of investigator-blind and independent data analysts.

## Conclusion

In conclusion, among patients who had received breast-conserving treatment for early-stage breast cancer, the rate of local recurrence was significantly higher for APBI than for WBI, but no differences in distant metastasis, breast cancer deaths, contralateral breast cancer, disease-free survival, and overall survival rates. Based on our preliminary investigation of radiotherapy techniques, we recommend that external beam-based APBI should be considered in the treatment choice for selected low-risk patients, that brachytherapy is only available in experienced institutions and intraoperative radiotherapy should be used with caution.

## Data Availability

All data generated or analyzed during this study are included in this manuscript.

## Abbreviations

IMRT: Intensity-modulated radiation therapy
IORT: Intraoperative irradiation
3DCRT: Three-dimensional conformal radiation therapy
EBRT: Whole-breast external beam radiotherapy
HDR: High dose rate
WBI: Whole-breast irradiation
APBI: Accelerated breast irradiation
ER: Estrogen receptor
HER2: Human epidermal growth factor receptor 2
IDC: Invasive duct carcinoma
DCIS: Ductal carcinoma in situ
ILC: Invasive lobular carcinoma
BID: Boost tumor bed, two fractions per day
NR: Not reported
OS: Overall survival
DFS: Disease-free survival
LR: Local recurrence
RR: Regional recurrence
NR: No reported
Adverse cosmesis: Physician-scored cosmetic results fair and poor
Second primary cancers: Had a second primary tumor in the contralateral-breast or a second tumor at a site other than the breast
SE: Standard error
